# The Influence of Verb-Based Implicit Causality Information and Second Language Proficiency on Chinese English Learners’ Pronoun Anaphoric Inference: Evidence from Eye Movements

**DOI:** 10.3390/bs14111034

**Published:** 2024-11-04

**Authors:** Huan Liu, Shifa Chen, Ruiyong Liu, Huinan Du

**Affiliations:** 1 College of Foreign Languages, Ocean University of China, Qingdao 266100, China; chenshifa99@ouc.edu.cn; 2 School of International Studies, Zhejiang University, Hangzhou 310058, China; lry8162@stu.ouc.edu.cn

**Keywords:** implicit causality, L2 proficiency, pronoun anaphoric inference, Chinese English learners, eye movements

## Abstract

Implicit causality (IC) is a phenomenon where verbs supply information about the potential cause of the behavior or state to one of the antecedents (e.g., “Mary praised David because…” will continue about David, not Mary). The study examines the influence of IC information and second language (L2) proficiency on Chinese English learners’ pronoun anaphoric inference. Results from an eye-tracking study showed that Chinese English learners can actively use IC information in making pronoun anaphoric inference. Additionally, compared to low-proficiency learners, high-proficiency learners spent less time on making pronoun anaphoric inference. The findings indicate that Chinese English learners can activate IC information early, before the disambiguating information appears, thus supporting the focusing account. Furthermore, L2 proficiency also affects this process.

## 1. Introduction

To comprehend a text, readers need to understand not only individual words but also the connection between linguistic units, which often span clauses or sentences [1]. Anaphoric inference plays an important role in this process. Anaphoric inference refers to the fact that a reader makes a connection between an anaphor and its referent or antecedent [2]. For instance, in the sentence “John praised Jack because he behaved well”, to determine the referent of the pronoun “he”, readers must make an anaphoric inference, linking “he” to “Jack” in the main clause. In the process of making such pronoun anaphoric inference, the verb plays a critical role. Readers use information implicitly encoded by the verb to construct event representations associated with causally related clauses [3]. In the above example, “he” refers to “Jack” because the verb “praise” in the main clause belongs to the agent–evocator (AE) verb class and according to Rudolph and Försterling [4], in sentences with AE verbs, the cause is mainly attributed to the object.

The phenomenon described above is known as implicit causality (IC), first identified by Garvey and Caramazza [5]. It refers to the information encoded in verbs that implicitly attributes the cause of an action or attitude to one of the antecedents mentioned earlier in the sentence. For example, in the sentence fragment, “John apologized to Mary because…”, readers tend to continue the sentence with a description related to the subject, regarding “John” as the cause of the “apologize to” event. Conversely, in the sentence fragment “John praised Mary because…”, readers typically continue with a description related to the object, viewing “Mary” as the underlying cause of the “praise” event. Thus, verbs can trigger inferences, and during this process, readers may assign the cause to the subject (the first noun phrase, NP1) or the object (the second noun phrase, NP2). When the cause is assigned to NP1, the verb is called an “NP1-biased verb”. When NP2 is seen as the underlying cause, the verb is referred to as an “NP2-biased verb”. However, in addition to the usual condition where the continuation is congruent with the verb bias (see Example 1a), the pronoun in the subordinate clause can also contradict the verb bias in the main clause (see Example 1b).

**Example 1a.**  Congruent: *John praised Mary because she was responsible for the successful arrangement.*

**Example 1b.**  Congruent: *Mary praised John because she was pleased with the successful arrangement.*

The pronoun in (Example 1b) is incongruent with the verb bias because the verb “praise” is NP2-biased, and under normal conditions, the pronoun should be assigned to the object. However, in (Example 1b), the pronoun refers to the subject, indicating that pronoun assignment can also be influenced by contextual information from the subordinate clause. Previous studies have shown that readers take longer to comprehend sentences where the pronoun in the subordinate clause is incongruent with the verb bias compared to sentences where the bias is congruent during the process of pronoun anaphoric inference [6,7,8,9,10,11,12].

In addition to the influence of verb-based IC information, individual differences such as reading skill [13], mood [14], working memory capacity [9,15], and language proficiency [16] play a significant role in pronoun anaphoric inference. The relationship between verb-based IC information and pronoun anaphoric inference is often modulated by a learner’s ability to process linguistic cues in real time, which can vary greatly depending on language proficiency. Specifically, L2 proficiency is critical for successful L2 processing, influencing not only the speed of lexical access and syntactic parsing but also the ability to integrate contextually relevant cues like verb-based IC information to make pronoun anaphoric inference.

In L2 acquisition, L2 learners often rely on different strategies from L1 learners when making pronoun anaphoric inference, because their processing system may not be fully optimized for the target language. L2 learners, particularly at lower proficiency levels, may face challenges in integrating verb-based IC cues due to limited exposure to the target language, reduced processing efficiency, and reduced predictive processing ability. Grüter et al. [17,18] introduced the Reduced Ability to Generate Expectations (RAGE) hypothesis as a way to explain how L2 learners differ from L1 learners in terms of their predictive processing abilities. Predictive processing is the brain’s capacity to anticipate upcoming linguistic information based on prior context, which helps facilitate faster and more efficient comprehension. In L1 learners, this ability to generate expectations plays a critical role in real-time sentence processing. L1 learners often rely on their extensive linguistic knowledge and experience to predict the next word or grammatical structure, which allows for more efficient processing and understanding of the sentence as a whole. However, according to the RAGE hypothesis [17,18], L2 learners tend to generate fewer and weaker expectations during sentence processing compared to L1 learners. This reduced ability can stem from several factors, such as limited linguistic exposure, heavier cognitive load, and weaker sensitivity to probabilistic cues. As a result, L2 learners may not assign the correct referent as readily or accurately as native speakers, particularly when complex syntactic or semantic cues, like verb-based IC information, are involved. This difference in processing becomes critical in contexts where verb-based IC information plays a primary role in making pronoun anaphoric inference, and it highlights the importance of considering L2 proficiency in studies of pronoun anaphoric inference. However, despite the increasing recognition of language proficiency as a key factor in L2 processing, relatively few studies have directly examined its influence on pronoun anaphoric inference, particularly within the framework of verb-based IC information. The existing literature has largely focused on native speakers or has not considered the interplay of verb-based IC information and L2 proficiency.

Furthermore, studies on languages with typological differences, such as Chinese and English, are scarce. Chinese, as a language that relies less on overt grammatical marking for referents and has certain language-specific features, such as verbal morphology and null reference, offers a unique context for exploring how L2 learners make pronoun anaphoric inference in English. This cross-linguistic context is important because L2 learners may draw on their native language strategies when processing English sentences, particularly in cases where IC biases might conflict with L1 referential practices. This choice allows us to examine how L1-specific properties might interact with the L2 processing of verb-based IC information, contributing to the broader understanding of verb-based IC phenomena across different languages. By focusing on L1-Chinese speakers, this study provides insight into how learners from a non-Indo-European linguistic background activate IC information in English, contributing to the understanding of L2 acquisition across diverse L1 backgrounds. Given the structural differences between Chinese and English, this study’s findings have implications not only for pronoun anaphoric inference research but also for broader questions of cross-linguistic influence and transfer in L2 processing.

Therefore, this research aims to deepen the understanding of how verb-based IC information and L2 proficiency jointly influence pronoun anaphoric inference in Chinese English learners. By investigating this relationship, the study not only addresses a critical gap in the literature but also contributes to broader discussions in L2 acquisition, specifically how L2 learners process IC information and integrate it with anaphoric cues. Additionally, the findings of this research have practical implications for language instruction and assessment, particularly for L2 learners of English, as it will shed light on the specific challenges they face in processing complex referential structures.

### 1.1. Verb-Based IC Information and Pronoun Anaphoric Inference

Comprehending sentences and discourse depends on the use of semantic, syntactic, pragmatic information, as well as world knowledge. Sentence and discourse processing requires readers to integrate various types of information to construct coherent discourse representations. Verb-based IC information is related to both verbal properties and discourse coherence, as it generates an expectation for an explanation [15,19]. Consequently, the influence of verb-based IC information on pronoun anaphoric inference has been extensively explored [10,12,20,21,22,23,24,25,26,27,28]. Numerous studies have demonstrated that L1 learners can actively use verb-based IC information to make pronoun anaphoric inference in both offline sentence completion and online sentence comprehension tasks [7,8,9,12,29]. In offline production tasks (e.g., “John praised Paul because he…”), readers tend to produce statements consistent with the verb bias when reading sentences with IC verbs in the main clause followed by a “because” subordinate clause [5,7,30]. In real-time sentence comprehension tasks, L1 learners can also employ verb-based IC information to make pronoun anaphoric inference, with the congruent condition requiring less processing time than the incongruent condition [11,31,32]. These studies indicate that verb-based IC information plays a crucial role for L1 learners in constructing coherent mental representations during pronoun anaphoric inference.

Although scholars have reached a consensus that verb-based IC information strongly affects pronoun anaphoric inference, the debate regarding the timing of its activation and utilization during this process has not been settled conclusively. Regarding this dispute, two main theories have been proposed: the focusing account and the integration account.

The focusing account [10,13,24,26,33] posits that verb-based IC information influences sentence processing very early, potentially as soon as the verb is encountered or at the onset of the subordinate clause. This theory suggests that during sentence comprehension, readers or listeners use the information provided by IC verbs to immediately focus their attention on a likely referent for subsequent pronouns. The theory holds that the verb triggers anticipatory processing, leading the reader or listener to predict the likely subject or object that will be mentioned next, especially in the context of a subordinate clause following the verb. This account emphasizes that readers or listeners use verb-based IC information to predict the upcoming referent. According to this theory, as soon as the reader or listener encounters an IC verb, they begin to anticipate the pronoun reference in the subsequent part of the sentence. For example, when a reader sees the verb “praise”, they expect that the object of the sentence will be the referent of the next pronoun. This proactive use of verb-based IC information facilitates more efficient sentence comprehension, as the processor has already focused on a specific entity before reaching the pronoun or the end of the clause. In summary, the focusing account highlights the proactive, predictive nature of verb-based IC information processing, where this information is used early to anticipate the correct referent of the pronoun even before the subordinate clause is fully processed.

In contrast, the integration account [12,32,34,35] argues that verb-based IC information does not affect sentence comprehension until later in the sentence, particularly after the entire subordinate clause has been processed and integrated. According to this theory, readers or listeners do not immediately use verb-based IC information when they encounter the verb. Instead, they wait until more disambiguating information is available, typically at the end of the clause, before they activate the IC bias and integrate it with the rest of the sentence. Unlike the focusing account, the integration account posits that verb-based IC information is not immediately activated upon encountering the verb. Instead, readers or listeners wait until they have received more information from the sentence—especially from the subordinate clause—before they begin to apply the IC biases from the verb. For example, even if a verb like “praise” suggests that the object is the likely referent of the pronoun, this information will not be used until the full clause has been read and processed. According to this theory, the pronoun reference is only resolved after the reader or listener has processed the entire subordinate clause. The reason for this delay is that verb-based IC information alone is not enough to determine the correct referent. Additional contextual information—such as other syntactic and semantic cues from the subordinate clause—must be integrated before the IC bias is fully applied. In brief, the integration account suggests that verb-based IC biases play a role after the complete subordinate clause has been processed and integrated into the overall meaning of the sentence. This account highlights the retrospective application of verb-based IC information, as readers rely on the totality of the clause to determine the appropriate pronoun reference.

Despite a lack of consensus on the exact time at which verb-based IC information is activated, increasing evidence indicates that verb-based IC information influences L1 learners’ pronoun anaphoric inference before the disambiguous information appears in the subordinate clause. However, what remains unclear is the effect of verb-based IC information on Chinese English learners’ pronoun anaphoric inference and whether this phenomenon extends to L2 learners in general.

For L2 learners, evidence is also from both offline and online studies. Offline studies typically use sentence completion tasks to investigate whether L2 learners are sensitive to verb-based IC information encoded in the verb [15,36,37,38]. Generally, L2 learners exhibit re-mention biases similar to those of L1 learners. Both L1 and L2 learners tend to produce more references to NP1 when the verb is NP1-biased and more references to NP2 when the verb is NP2-biased. In online studies, evidence also indicates that verb-based IC information impacts L2 learners’ pronoun anaphoric inference [15,37,38,39,40]. Researchers have adopted the self-paced reading task to investigate Chinese English learners’ activation of verb-based IC information in English pronoun anaphoric inference. These studies have found significant time slowdowns when participants read sentences in the incongruent condition compared to the congruent condition under both NP1-biased and NP2-biased conditions [15,39,40]. This suggests that Chinese English learners are sensitive to verb-based IC information and can utilize it for pronoun anaphoric inference. More recent studies have employed the visual world paradigm in eye-tracking to explore whether verb-based IC information can be used proactively during the process of pronoun anaphoric inference [3,37,38]. In light of Contemori and Dussias’s observation [37], verb-based IC information is only activated on the NP1-biased condition, and compared to monolinguals, Spanish–English bilinguals showed a nearly 400ms delay. However, reanalysis by Contemori and Dussias [38] found similar performance between the two groups in the NP1-biased condition, indicating that highly proficient bilinguals processed information similarly to monolinguals, with no significant delay. Using the same paradigm, Kim and Grüter [3] studied Korean English learners’ use of verb-based IC information and found that both L1 and L2 learners could use verb-based IC information at an early time, but this effect was weaker and appeared later for L2 learners.

Although related studies have suggested that L2 learners can make use of the semantic information provided by the IC verb to make pronoun anaphoric inference, evidence concerning the activation of verb-based IC information in L2 learners’ online pronoun anaphoric inference is insufficient, and results from aforementioned studies about L2 learners are not fully conclusive [1,3,15,16,36,37,38,39]. Furthermore, it is crucial to consider that L2 learners may face distinct challenges when processing IC information. For example, L2 learners might activate IC information later or less efficiently than L1 learners due to lower proficiency or limited exposure to naturalistic discourse. This aligns with findings from the Reduced Ability to Generate Expectations (RAGE) hypothesis [17,18], which posits that L2 learners generate fewer and weaker expectations during sentence processing than L1 learners. The RAGE hypothesis suggests that L2 learners, particularly those learning in non-immersive environments, may struggle with predictive processing, which could result in delayed or reduced use of verb-based IC information. Moreover, previous studies have tested the focusing and integration accounts in L1 learners, but fewer have explored their applicability to L2 processing, particularly for Chinese English learners. Thus, Chinese English learners’ activation of verb-based IC information needs to be further explored.

### 1.2. Individual Differences and Pronoun Anaphoric Inference

There is a growing awareness that individual differences also influence pronoun anaphoric inference in sentences with verb-based IC information. Factors such as reading skill [13], mood [14], working memory capacity [9,15], language proficiency [16], print exposure [41], and vocabulary knowledge [15] have been identified as significant influences.

Long and De Ley [13] adopted the probe recognition paradigm to examine how pronoun anaphoric inference is influenced by verb-based IC information and the role of readers’ reading skills in this process. Their investigation revealed that only skilled readers exhibited an early IC effect, responding more quickly to names that matched the verb bias compared to names that did not. In contrast, less skilled readers were influenced by verb-based IC information only at the end of the sentence, when integrating information from both clauses. Moreover, the effect of verb-based IC information observed in skilled readers was limited to NP2-biased verbs. Furthermore, Van Berkum et al. [14] proposed that readers’ moods could also influence the usage of verb-based IC information in pronoun anaphoric inference through an event-related potentials (ERPs) study. They found that only when in a good mood, subjects displayed signs of activating verb-based IC information, and the influence of this information was absent for readers in a bad mood.

In investigations by Koornneef et al. [9] and Wang and Gabriele [15], the influence of working memory on the use of verb-based IC information was considered. Koornneef et al. [9] employed the normal sentence reading paradigm combined with forward and backward digit span tasks to explore Dutch speakers’ utilization of verb-based IC information in pronoun anaphoric inference. Their findings were somewhat complex. Without an additional working memory task, results aligned with expectations; readers with higher working memory capacity were more likely to use verb-based IC information, with significant differences between high- and low-memory-span readers observed at the critical pronoun and three words following it. However, when participants were required to memorize and recall a sequence of digits, the pattern reversed. Under memory pressure, lower-span readers used verb-based IC information more proactively than higher-span readers, with significant differences emerging three words after the critical pronoun. This suggested that the activation of verb-based IC information was not inherently resource-consuming. In contrast, Wang and Gabriele [15] examined the sensitivity to IC bias among English natives and Chinese English learners using the self-paced reading paradigm and the counting span task. Their findings indicated that the activation of verb-based IC information is a resource-consuming mental process. In their experiments, readers with higher working memory capacity had more resources available to integrate verb-based IC information immediately after the pronoun.

Furthermore, Wang and Gabriele [15] investigated the influence of vocabulary knowledge on pronoun anaphoric inference in sentences with verb-based IC information. Their study demonstrated that, in spillover regions, learners with higher vocabulary scores were more likely to employ verb-based IC information in sentences with both NP1-biased and NP2-biased verbs. This effect was observed for both L1 and L2 learners with higher vocabulary scores. Additionally, Kim and Grüter [3] conducted a visual world eye-tracking experiment to examine the use of verb-based IC information in the inference process of Korean English learners with varying L2 proficiency. Their results showed that both L1 and L2 learners could access and utilize this information before the disambiguating information emerged, although the L2 group exhibited a somewhat slower rate and lesser extent compared to the L1 group. No evidence was found for L2 proficiency as an influencing factor in this study. Moreover, Johnson and Arnold [41] explored the impact of print exposure on pronoun comprehension in sentences with verb-based IC information. Participants listened to an audio recording, viewed pictures of the mentioned characters, and answered two multiple-choice comprehension questions. Their results indicated that participants with higher scores demonstrated a stronger semantic bias than those with lower scores.

Moreover, Kaan [42] explored predictive sentence processing in L2 learners and found key differences between L1 and L2 speakers regarding expectation generation. Kaan noted that L2 learners, compared to native speakers, tend to have weaker and less consistent expectations due to additional cognitive demands and differences in proficiency. This is particularly relevant when considering the role of verb-based IC information in making pronoun anaphoric inference, as Kaan’s findings suggest that L2 learners may struggle to generate and maintain verb-based expectations, potentially leading to more variability in pronoun anaphoric inference.

In summary, studies have shown that individual differences can affect pronoun anaphoric inference in sentences with verb-based IC information. Therefore, the impact of individual differences should be considered in related research. Additionally, predictive behaviors are expected to increase with higher proficiency [42,43,44,45]. However, Kim and Grüter [3] did not find evidence for L2 proficiency as an influencing factor, suggesting the need for further research. Furthermore, advanced English learners in studies by Contemori and Dussias [37,38] and Liu and Nicol [39] had varying years of formal education or residence in English-speaking countries. It remains unclear how Chinese English learners with different L2 proficiency levels, particularly those who do not learn English in an immersive environment and only use it in class, utilize verb-based IC information in English pronoun anaphoric inference during online sentence processing. Thus, this study aims to fill this gap by exploring how non-immersed Chinese English learners with varying L2 proficiency use verb-based IC information to make English pronoun anaphoric inferences using a normal sentence reading paradigm with eye movements. Specifically, the research seeks to determine whether verb-based IC information affects Chinese English learners’ pronoun anaphoric inference and when this information is activated, as well as the influence of L2 proficiency on this process. Additionally, to provide a clearer understanding of whether verb-based IC information operates differently in L2 processing, this study will test how the focusing and integration account apply to L2 learners and whether these accounts make different predictions for L1 versus L2 learners, while considering the individual differences and potential contributions of the RAGE hypothesis to predictive processing in L2 learners.

Based on these objectives, the hypotheses are as follows. Firstly, according to previous studies [7,10,12], if verb-based IC information is activated, participants should exhibit slower responses in the incongruent condition compared to the congruent condition. For L1 learners, if the delay occurs at or shortly after the pronoun, it supports the focusing account. Conversely, the integration account predicts that the delay will happen at the end of the subordinate clause. For L2 learners, these predictions may differ. The RAGE hypothesis suggests that L2 learners may not activate verb-based IC information as early as L1 learners, which would align more with the integration account. Alternatively, highly proficient L2 learners might behave similarly to L1 learners, supporting the focusing account. Secondly, based on related research [13,15], L2 proficiency should affect pronoun anaphoric inference in sentences with verb-based IC information, with the higher proficiency group more likely to utilize verb-based IC information and requiring less processing time than the lower proficiency group.

## 2. Methods

### 2.1. Participants

A total of 83 Chinese English learners (24 male, mean age = 22, age range: 18–27) were recruited from a university in China, and they received awards for their participation. All participants were native Chinese speakers whose second language was English. They began learning English in school between the ages of 9 and 12. None of the participants had visited an English-speaking country. They had normal or corrected-to-normal vision and no diagnosed reading or learning disabilities. None of them had participated in a normality test. Before the eye movement experiment, participants signed an informed consent form and took the Oxford Placement Test (OPT). Based on the test results, 9 participants were excluded due to middling and low scores. The remaining 74 participants were divided into two groups according to their grades; 40 participants were placed in the high-proficiency group (8 male, mean grade = 82.0, *SD* = 5.3, grade range: 75–93) and 34 participants in the low-proficiency group (15 male, mean grade = 62.3, *SD* = 5.4, grade range: 51–69). An independent-sample t-test revealed a significant difference in L2 proficiency between the two groups (*p* < 0.001).

All of them were Chinese native speakers whose L2 was English. They began to study English in school at ages from 9 to 12 years old. None of them had been to an English-speaking country. They had normal or corrected to normal vision and were without a diagnosed reading or learning disability. None of them took part in the normality test. Before the eye movement experiment, they signed the informed consent and took part in the Oxford Placement Test (OPT). After this test, 9 participants were eliminated due to middling and low grades. According to the grade, the remaining 74 participants were divided into two groups: 40 in the high-proficiency group (8 male, mean grade = 82.0, *SD* = 5.3, grade range: 75–93) and 34 in the low-proficiency group (15 male, mean grade = 62.3, *SD* = 5.4, grade range: 51–69). An independent-sample t-test was conducted to compare the proficiency of the two groups. The result showed that there was a significant difference in L2 proficiency between the two groups (*p* < 0.001).

### 2.2. Materials

The stimuli consisted of 112 sentences, made up of 56 experimental sentences and 56 filler sentences. The experimental sentences were based on 28 verbs, with 14 NP1-biased verbs and 14 NP2-biased verbs. Each verb appeared in two versions, categorized by congruency. All sentences were structured with a main clause and a subordinate clause connected by “because”. The main clause featured two proper English names of opposite genders, which served as the subject and object of an active past tense verb. For half of the sentences, the subject was female, and for the other half, it was male. Gender was also balanced across NP1 and NP2 sentences. The subordinate clause began with a pronoun subject. The critical manipulation involved whether the pronoun was congruent with the verb bias. The pronoun remained constant across conditions, while the bias manipulation was achieved by altering the order of the two names in the main clause. One name was male, and the other was female. This manipulation method mirrored that used in previous studies [7,10]. For example, participants might read sentences such as (Example 2a) and (Example 2b) with an NP1-biased verb or (Example 3a) and (Example 3b) with an NP2-biased verb. The continuation could either be congruent with the verb bias, as illustrated in (Example 2a) and (Example 3a), or incongruent, as shown in (Example 2b) and (Example 3b). To control for processing strategies, 56 filler sentences were included, which did not exhibit a clear verb bias and employed various connectives (e.g., before, after, although, while, and, but) or no connective.

**Example 2a.**  NP1-biased verb, bias-congruent pronoun: *John annoyed Mary because he kept making the same mistake while practicing.*

**Example 2b.**  NP1-biased verb, bias-incongruent pronoun: *Mary annoyed John because he hated people talking about his past life.*

**Example 3a.**  NP2-biased verb, bias-congruent pronoun: *John admired Mary because she could speak six foreign languages at a young age.*

**Example 3b.**  NP2-biased verb, bias-incongruent pronoun: *Mary admired John because she liked people with exploration and innovation spirit.*

The construction of experimental stimuli involved several detailed steps. Materials were selected through a series of normality tests, including assessments of verb familiarity, verb bias, English name familiarity and gender typicality, as well as sentence plausibility.

**Verb familiarity test**. This study evaluated 305 candidate verbs, drawing from the verb bias corpus in English provided by Ferstl et al. [30] and Garnham et al. [46]. Thirty-nine sophomores from a university in China were recruited to rate the familiarity of these verbs on a scale from 1 (very unfamiliar) to 7 (very familiar) (Cronbach’s alpha = 0.990). Verbs with a familiarity rating greater than four were selected, resulting in a total of 179 verbs: 2 with no bias, 89 NP1-biased verbs (familiarity: *M* = 5.6, *SD* = 0.7, range: 4.0–6.6), and 88 NP2-biased verbs (familiarity: *M* = 5.6, *SD* = 0.7, range: 4.0–6.6). A paired-sample t-test was conducted to examine whether verb familiarity differed between NP1-biased and NP2-biased verbs. The results indicated no statistically significant difference in familiarity between the two verb types (*t* = −0.432, *p* = 0.673), suggesting that verb familiarity did not influence the outcomes.

**Verb bias test**. From the initial set of 89 NP1-biased and 88 NP2-biased verbs, 50 NP1-biased verbs (familiarity: *M* = 5.6, *SD* = 0.6, range: 4.4–6.5; bias: *M* = 68.5, *SD* = 11.7, range: 47–93) and 50 NP2-biased verbs (familiarity: *M* = 5.6, *SD* = 0.7, range: 4.1–6.6; bias: *M* = −75.7, *SD* = 14.0, range: −50–−98) with clear bias were selected. To ensure that these verbs were also strongly biased for L2 learners, 66 juniors from a university in China evaluated the bias of these 100 verbs. Given the limited English production abilities of L2 learners, this study deviated from the sentence completion task used in previous experiments [10,27,36,47] and instead asked participants to directly judge the bias of these verbs within sentence fragments. Participants were required to decide whether the continuation of a sentence fragment referred to one of the two referents mentioned or not. Each fragment included a verb, two gender-different English proper names as the subject and object, and the connective “because”. Verbs were presented in the past tense, e.g., “Anna praised Tom because…”. The order of male and female names was counterbalanced. According to Koorneef and Berkum’s [10] scoring criteria, if the continuation referred to the subject, it was marked as “1”; if it referred to the object, it was marked as “2”. Other cases, such as plural references (e.g., they) or no reference to either referent, were marked as “1.5”. Based on these evaluations, 14 NP1-biased verbs (bias: *M* = 1.28, *SD* = 0.07, range: 1.16–1.36; familiarity: *M* = 5.8, *SD* = 0.5, range: 4.9–6.5) and 14 NP2-biased verbs (bias: *M* = 1.82, *SD* = 0.06, range: 1.74–1.95; familiarity: *M* = 5.9, *SD* = 0.5, range: 4.6–6.5) were selected as the critical verbs for this study. Detailed information about these verbs is presented in Table 1.

**English name familiarity and gender typicality test**: Given that Chinese English learners might not be very familiar with English names, assessing the gender of these names could be challenging. Therefore, this test aimed to determine whether the names used in this experiment were familiar to Chinese English learners and whether they were perceived as typically male or female. The selection process was as follows. First, 109 commonly used English names from Chinese middle school English textbooks were collected, comprising 54 male and 55 female names. Second, 69 juniors from a university in China were asked to rate the familiarity and gender typicality of these names on a seven-point scale (familiarity: “1” representing extremely unfamiliar and “7” representing extremely familiar; typicality: “1” representing typically male and “7” representing typically female), with 34 students completing Questionnaire I (Cronbach’s alpha = 0.970 for familiarity; Cronbach’s alpha = 0.791 for gender typicality) and 35 students completing Questionnaire II (Cronbach’s alpha = 0.966 for familiarity; Cronbach’s alpha = 0.954 for gender typicality). Third, based on the 28 selected verbs from the previous test, 28 pairs of English names were chosen: 28 high-familiarity typical male names (familiarity: *M* = 6.2, *SD* = 0.6, range: 4.4–6.9; typicality: *M* = 2.3, *SD* = 0.1, range: 1.9–2.5) and 28 high-familiarity typical female names (familiarity: *M* = 6.3, *SD* = 0.4, range: 5.4–6.8; typicality: *M* = 6.4, *SD* = 0.3, range: 6.1–6.9).

**Sentence plausibility test**. Based on the 28 selected verbs, sentences with plausible semantics were chosen from the sentence completion task results of English native speakers [30]. These sentences were partially modified to ensure grammatical correctness and semantic plausibility. To prevent strategic processing, two questionnaires were designed, each containing 28 sentences. Each questionnaire included an equal number of sentences in the congruent and incongruent conditions. Fifty-four students from a university in China rated the sentences for plausibility on a scale from 1 (very implausible) to 7 (very plausible), with 29 sophomores completing Questionnaire I (Cronbach’s alpha = 0.923) and 25 sophomores completing Questionnaire II (Cronbach’s alpha = 0.970). All sentences were rated as highly plausible, with average scores of 6.5 for Questionnaire I and 6.4 for Questionnaire II. For the NP1- and NP2-biased verbs, the congruent conditions had average scores of 6.5, while the incongruent conditions had average scores of 6.3 and 6.4, respectively. An independent-samples t-test revealed a significant difference in plausibility between the congruent and incongruent conditions (*p* = 0.016), indicating that readers found it more difficult to process incongruent sentences than congruent ones.

### 2.3. Procedure

A desk-mounted Eye-Link 1000 Plus system was used to monitor eye movements in this study. Eye movements were recorded from the right eye at a sampling rate of 1000 Hz. Participants were tested individually in a soundproof laboratory at a university in China. They sat in a chair of appropriate height, positioned 75 cm from the computer screen, with their heads stabilized by a chin and forehead rest. A three-point calibration was performed at the beginning of the experiment.

Participants received both verbal and written instructions to read sentences and answer questions. The experiment commenced with four practice trials designed to familiarize participants with the task. Following the practice trials, experimental sentences were displayed in Times New Roman, 30-point font, with each sentence appearing on a single line at the left center of the screen. Before each sentence appeared, a small black square fixation mark was shown at the position where the first word of the sentence would appear, signaling the start of the sentence. Participants were instructed to fixate on the mark, and the sentence would be presented after successful fixation and correction for drift. Upon completing the reading of a sentence, participants pressed either the “F” or “J” key, with key assignment counterbalanced between subjects. At that point, participants either saw a question or the fixation mark for the next trial.

To ensure comprehension, half of the sentences were followed by a two-choice question that did not directly ask what the pronoun referred to (e.g., “Did Frank praise Maria?”; see Koornneef et al. [10]). Questions were evenly distributed between experimental and filler sentences, and the correct answers were equally split between “Yes” and “No”. For “Yes” responses, participants pressed the “F” key with the index finger of their left hand; for “No” responses, they pressed the “J” key with the index finger of their right hand. The left and right hand were cross-balanced between subjects.

To minimize fatigue, stimuli were divided into two blocks, each containing 14 NP1-biased and 14 NP2-biased verbs, with 14 bias-congruent and 14 bias-incongruent conditions. Each block contained only one version of each sentence. Additionally, each block included 28 filler sentences, presented randomly. Participants took a 5–10 min break after completing half of the trials. Following the break, the next block began with recalibration. The entire experiment lasted approximately 40 min.

### 2.4. Data Analysis

Data were extracted using Data Viewer software. Before conducting any analyses, participants’ performance on the comprehension questions was evaluated. Data from three participants were excluded: one due to inattentiveness, one due to excessive blinking, and one due to a high error rate (exceeding 30%). The remaining 71 participants (38 high-proficiency and 33 low-proficiency) had an average accuracy of 93.2% (*SD* = 4.1), indicating that they generally performed well on the task. Major tracker losses and eye blinks were addressed. Fixations shorter than 80 ms or longer than 1200 ms were discarded as such durations were considered unrepresentative of reading processing. Finally, reading times exceeding 2.5 standard deviations from the mean for any eye movement measure within a particular region and condition were treated as missing data. Based on these data selection criteria, outliers comprised 3.0% of the data in the critical pronoun region and 2.8% in the critical region.

In accordance with Koornneef and Sanders [47] and Sun et al. [29], this study focused on areas of interest, including the critical pronoun in the subordinate clause and the critical region “because + the pronoun”. The selected eye movement measures were based on those used in the aforementioned studies and included both early measures (first fixation durations, FFD; and first gaze durations, FGD) and late measures (regression path durations, RPD; and total reading times, TRT). FFD represents the duration of the very first fixation on the current word. FGD is the sum of all fixation durations on the current word before a reader moves on or looks back in the sentence. RPD encompasses all fixation durations and saccades from the moment a reader encounters a word until they enter a subsequent region, including any regressions. That is, if a reader looks back after reading a particular region, the regression path time contains all fixation and saccade durations of this regression. TRT is the total duration of all fixations within the current area of interest.

To stabilize variance and approximate a normal distribution, the data were log-transformed prior to analysis [48]. Data were analyzed using the LME4 package in R (version 4.2.1) [49]. The independent variables included verb bias (NP1 versus NP2), congruency (congruent versus incongruent), and L2 proficiency (high versus low). The dependent variables were the four eye movement measures: FFD, FGD, RPD, and TRT. Subjects and items were treated as random effects, while verb bias, congruency, and L2 proficiency were treated as fixed effects in the linear mixed-effects model. Models were initially fitted with the maximal random effects structure; if convergence issues occurred, random slopes were progressively removed. The most appropriate models were identified using Akaike Information Criterion (AIC) values. When significant interactions among verb bias, congruency, and L2 proficiency were detected, the emmeans package [50] was used for simple effects analysis.

## 3. Results

**Figure 1:**
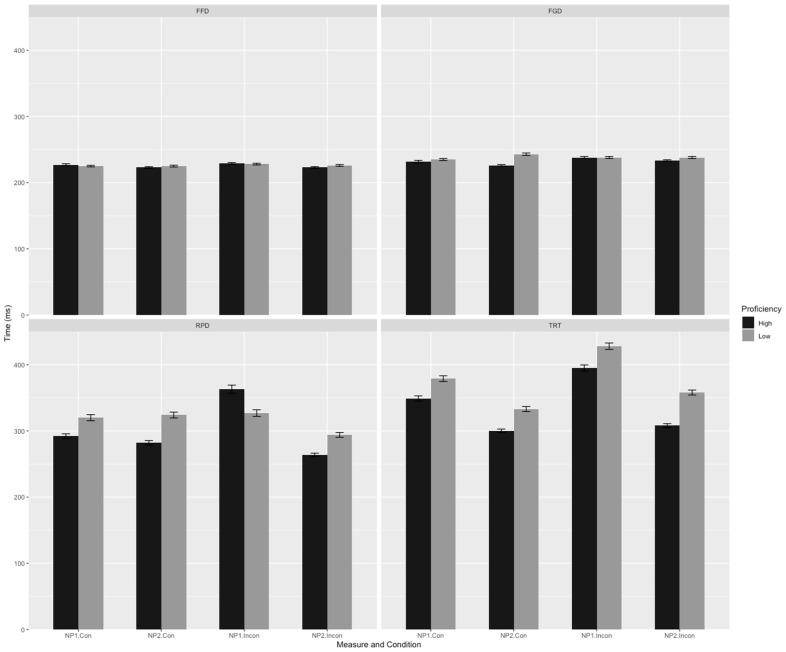
Mean reading times of different eye movement measures at the critical pronoun. Note: (1) FFD: first fixation durations, FGD: first gaze durations, RPD: regression path durations, TRT: total reading times; Con.: congruent, Incon.: incongruent. (2) To display participants’ reading times more intuitively, the data in the figure are presented as raw data.

**Figure 2:**
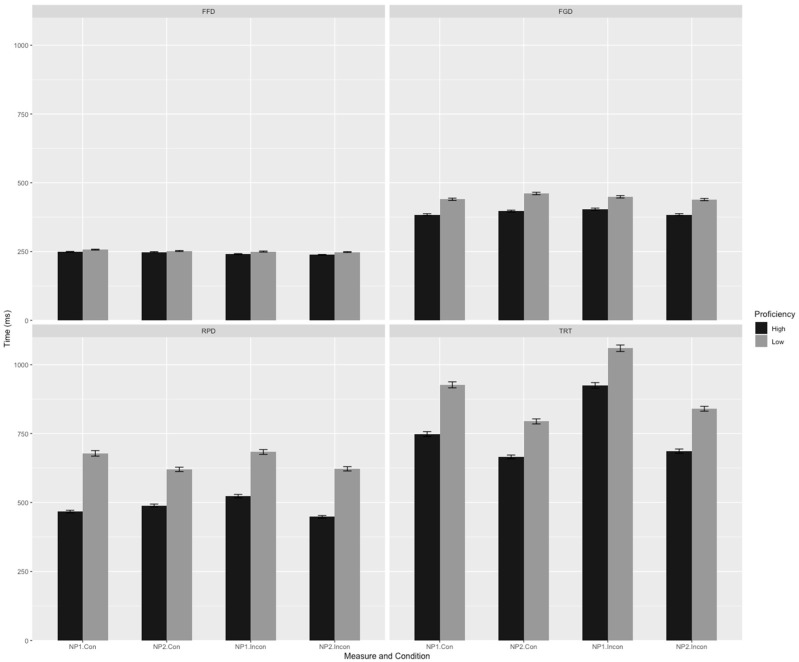
Mean reading times of different eye movement measures at the critical region. Note: (1) FFD: first fixation durations, FGD: first gaze durations, RPD: regression path durations, TRT: total reading times; Con.: congruent, Incon.: incongruent. (2) To display participants’ reading times more intuitively, the data in the figure are presented as raw data.

Mean reading times and standard deviations for various eye movement measures at both the critical pronoun and the critical region are presented in Figure 1 and Figure 2.

Figure 1 and Figure 2 present the descriptive statistics for different eye movement measures. A linear mixed-effects model was employed to analyze the data of the four eye movement measures at the critical pronoun and critical region, focusing on the effects of verb bias, congruency, L2 proficiency, and their interactions. The results of the detailed statistical analysis are as follows.

### 3.1. Critical Pronoun

Table 2 summarizes the output of different eye movement measures at the critical pronoun. The results indicate that there was a significant main effect of verb bias on total reading times (*β* = −0.125, *SE* = 0.046, *t* = −2.709, *p* = 0.007, *η*^2^ = 0.150), with the NP2-biased condition requiring less processing time than the NP1-biased condition. In addition, the main effect of congruency was significant on both regression path durations (*β* = 0.141, *SE* = 0.037, *t* = 3.756, *p* < 0.001, *η*^2^ = 0.006) and total reading times (*β* = 0.110, *SE* = 0.044, *t* = 2.494, *p* = 0.013, *η*^2^ = 0.030). On both eye movement measures, participants processed the congruent condition more quickly than the incongruent condition.

The effect of the two-way interactions between L2 proficiency and congruency (*β* = −0.134, *SE* = 0.053, *t* = −2.528, *p* = 0.012, *η*^2^ = 0.002) and between verb bias and congruency (*β* = −0.170, *SE* = 0.054, *t* = −3.156, *p* = 0.002, *η*^2^ = 0. 080) were significant on regression path durations. Regarding the interaction between L2 proficiency and congruency, the simple effect analysis revealed that for the congruent condition, high-proficiency participants required significantly less time to process sentences than low-proficiency participants (*β* = −0.088, *SE* = 0.035, *t* = −2.516, *p* = 0.012). Under the incongruent condition, no significant difference in processing times was found between high and low-proficiency participants (*β* = −0.007, *SE* = 0.035, *t* = −0.197, *p* = 0.844). High-proficiency participants processed congruent sentences significantly faster than incongruent ones (*β* = −0.055, *SE* = 0.028, *t* = −1.994, *p* = 0.047), whereas low-proficiency participants showed no significant difference in processing times between congruent and incongruent sentences (*β* = 0.026, *SE* = 0.028, *t* = 0.914, *p* = 0.361).

For the interaction between verb bias and congruency, the simple effect analysis indicated that under the congruent condition, the difference between NP1-biased and NP2-biased verbs was not significant (*β* = 0.0002, *SE* = 0.029, *t* = 0.008, *p* = 0.994). However, under the incongruent condition, there existed significant differences between NP1-biased and NP2-biased verbs (*β* = 0.118, *SE* = 0.029, *t* = 4.150, *p* < 0.001). Specifically, when the verb was NP1-biased, readers spent more time on pronoun anaphoric inference than when the verb was NP2-biased. Regarding verb bias, if the verb was NP1-biased, the difference between congruent and incongruent conditions was significant (*β* = −0.074, *SE* = 0.029, *t* = −2.555, *p* = 0.012), with less processing time needed for the congruent condition than the incongruent condition. If the verb was NP2-biased, no significant difference between congruent and incongruent conditions was observed (*β* = 0.044, *SE* = 0.029, *t* = 1.521, *p* = 0.130).

### 3.2. Critical Region

Table 3 presents the results of various eye movement measures at the critical region. The data demonstrated that the main effect of L2 proficiency was apparently significant on first gaze durations (*β* = 0.122, *SE* = 0.045, *t* = 2.735, *p* = 0.007, *η*^2^ = 0.070), regression path durations (*β* = 0.312, *SE* = 0.054, *t* = 5.775, *p* < 0.001, *η*^2^ = 0.190), and total reading times (*β* = 0.220, *SE* = 0.064, *t* = 3.428, *p* < 0.001, *η*^2^ = 0.070). All low-proficiency readers spent more time making pronoun anaphoric inference than high-proficiency readers. In addition, there was a significant main effect of verb bias on total reading times (*β* = −0.114, *SE* = 0.037, *t* = −3.086, *p* = 0.002, *η*^2^ = 0.110), with the NP1-biased verb requiring more cognitive resources for pronoun anaphoric inference than the NP2-biased verb. Moreover, the main effect of congruency was observed on regression path durations (*β* = 0.096, *SE* = 0.036, *t* = 2.672, *p* = 0.008, *η*^2^ = 0.004) and total reading times (*β* = 0.233, *SE* = 0.039, *t* = 5.952, *p* < 0.001, *η*^2^ = 0.050), indicating that congruent sentences were easier to understand than incongruent sentences.

Moreover, the effect of the two-way interaction between L2 proficiency and verb bias (*β* = −0.105, *SE* = 0.047, *t* = −1.397, *p* = 0.026, *η*^2^ = 0.000) and between verb bias and congruency (*β* = −0.169, *SE* = 0.049, *t* = −3.422, *p* < 0.001, *η*^2^ = 0.020) on regression path durations was significant. Regarding the interaction between L2 proficiency and verb bias, the simple effect analysis revealed that irrespective of whether the verb was NP1-biased or NP2-biased, the difference between high- and low-proficiency groups was significant (*β* = −0.279, *SE* = 0.049, *t* = −5.707, *p* < 0.001; *β* = −0.249, *SE* = 0.049, *t* = −5.085, *p* < 0.001). High-proficiency participants needed less processing time than low-proficiency participants under both NP1-biased and NP2-biased conditions. From the perspective of L2 proficiency, the high-proficiency group showed a marginal slowdown in processing time during the NP1-biased condition compared to the NP2-biased condition (*β* = 0.046, *SE* = 0.025, *t* = 1.8345, *p* = 0.067). For the low-proficiency group, there was an apparent difference between the NP1-biased and NP2-biased verb (*β* = 0.076, *SE* = 0.027, *t* = 2.855, *p* = 0.004), with more processing time required for NP1-biased verbs. In terms of the interaction between verb bias and congruency, the simple effect analysis showed that under the congruent condition, the difference between NP1-biased and NP2-biased verbs was not significant (*β* = 0.014, *SE* = 0.028, *t* = 0.511, *p* = 0.610). However, under the incongruent condition, there was a significant difference between NP1-biased and NP2-biased verbs (*β* = 0.108, *SE* = 0.028, *t* = 3.849, *p* < 0.001), with NP1-biased verbs requiring more time. Additionally, for NP1-biased verbs, the difference between congruent and incongruent conditions was significant (*β* = −0.063, *SE* = 0.029, *t* = −2.129, *p* = 0.035), with the congruent condition requiring less time. Conversely, for NP2-biased verbs, no significant difference was found between congruent and incongruent conditions (*β* = 0.031, *SE* = 0.028, *t* = 1.126, *p* = 0.261).

Additionally, the effect of two-way interaction between verb bias and congruency on total reading times was significant (*β* = −0.219, *SE* = 0.052, *t* = −4.183, *p* < 0.001, *η*^2^ = 0.010). The simple effect analysis revealed that, regardless of whether the condition was congruent or incongruent, there was a significant difference between NP1-biased and NP2-biased verbs (congruent: *β* = 0.144, *SE* = 0.031, *t* = 4.704, *p* < 0.001; incongruent: *β* = 0.292, *SE* = 0.031, *t* = 9.452, *p* < 0.001). Participants processed NP2-biased conditions more easily than NP1-biased conditions. When the verb was NP1-biased, the difference between congruent and incongruent conditions was significant (*β* = −0.193, *SE* = 0.033, *t* = −5.769, *p* < 0.001). However, for NP2-biased verbs, no significant difference was found between congruent and incongruent conditions (*β* = −0.045, *SE* = 0.030, *t* = −1.502, *p* = 0.134).

Finally, the effect of the three-way interaction among L2 proficiency, verb bias and congruency showed significant differences on regression path durations (*β* = 0.150, *SE* = 0.067, *t* = 2.247, *p* = 0.025, *η*^2^ = 0.001) and total reading times (*β* = 0.143, *SE* = 0.066, *t* = 2.167, *p* = 0.030, *η*^2^ = 0.001). The detailed effect of the three-way interaction on regression path durations was as follows. First, irrespective of verb bias (NP1-biased or NP2-biased) or congruency (congruent or incongruent), the difference between high- and low-proficiency groups was significant (*ps* < 0.001), with the high-proficiency group consistently taking less time than the low-proficiency group. Second, from the perspective of L2 proficiency and congruency, the difference between NP1-biased and NP2-biased verbs was significant only under the incongruent condition (*ps* < 0.05), not under the congruent condition (*ps* > 0.05). NP1-biased verbs required more processing time than NP2-biased verbs. Third, from the perspective of L2 proficiency and verb bias, significant differences in processing time between congruent and incongruent conditions were observed only for the high-proficiency group (NP1-biased: *β* = −0.096, *SE* = 0.036, *t* = −2.621, *p* = 0.009; NP2-biased: *β* = 0.073, *SE* = 0.035, *t* = 2.084, *p* = 0.038) but not for the low-proficiency group (NP1-biased: *β* = −0.030, *SE* = 0.039, *t* = −0.759, *p* = 0.448; NP2-biased: *β* = −0.011, *SE* = 0.038, *t* = −0.288, *p* = 0.773). For NP1-biased verbs, high-proficiency readers required less time in the congruent condition than in the incongruent condition. Conversely, for NP2-biased verbs, high-proficiency readers required more time in the congruent condition than in the incongruent condition. On total reading times, further analysis showed that the high-proficiency group consistently required less time to process pronoun anaphoric inference than the low-proficiency group (*ps* < 0.05), regardless of verb bias or congruency. Additionally, participants took longer to process sentences containing NP1-biased verbs than NP2-biased verbs (*ps* < 0.05). For NP1-biased verbs, both the two groups took less time to process the congruent condition than the incongruent condition (*ps* < 0.05). However, for NP2-biased verbs, the difference between congruent and incongruent conditions was not significant for the high-proficiency group (*β* = −0.014, *SE* = 0.037, *t* = −0.371, *p* = 0.711) but was marginally significant for the low-proficiency group (*β* = −0.076, *SE* = 0.039, *t* = −1.951, *p* = 0.051).

In summary, the results revealed statistically significant effects of the three influencing factors—verb bias, congruency, and L2 proficiency—along with their interactions at the critical pronoun and critical region on various eye movement measures in this experiment. Detailed statistical analysis results are provided in Table 2 and Table 3.

## 4. Discussion

### 4.1. The Influence of Verb-Based IC Information

In the present study, the influence of verb bias was evident on TRT at both the critical pronoun and critical region. The results indicated that verb bias significantly impacted Chinese English learners’ pronoun anaphoric inference. Specifically, sentences with NP2-biased verbs were processed more quickly than those with NP1-biased verbs. This finding aligns with previous research by Lyu and Wang [11] and Stewart et al. [12]. Lyu and Wang [11] investigated the effect of verb-based IC information on Chinese pronoun anaphoric inference using comprehension questions following auditory sentence presentations. Their results revealed that sentences with NP2-biased verbs were processed significantly faster than those with NP1-biased verbs. Similarly, Stewart et al. [12] adopted a self-paced reading task with English native speakers and found that NP2-biased verbs facilitated faster reading than NP1-biased verbs.

This study’s results contrast with the first mention or subject preference account [51,52,53,54], which posits that the first-mentioned noun phrase is more accessible and thus more likely to be the preferred antecedent for a pronoun. According to this account, readers should have taken less time to resolve pronouns in NP1-biased conditions, as the first-mentioned noun phrase is considered more accessible. However, our findings suggest otherwise. Instead, our results support the distance effect [55], which asserts that pronouns are resolved more quickly when their antecedents are nearer. In our study, the NP2-biased noun phrase, being more recent, likely remains more salient in the readers’ short-term memory. Thus, Chinese English learners appear to benefit from the recency effect, making the most recent noun phrase more likely to be selected as the antecedent of a pronoun. Consequently, readers took less time to process pronouns in the NP2-biased condition than in the NP1-biased condition, reflecting a preference for the more recent referent.

Additionally, the results demonstrated that the difference between congruent and incongruent conditions was significant on RPD and TRT at both the critical pronoun and critical region. To be specific, sentences with incongruent conditions required more processing effort than congruent conditions. This finding aligns with the notion that, when verb-based IC information is activated, readers require more time to resolve pronouns in incongruent contexts than congruent ones. The significant difference in activation time between congruent and incongruent conditions at the critical pronoun and critical region indicates that this time delay occurs before the presentation of any disambiguating information. This result is strongly consistent with the focusing account [10,13,24,26,33], which posits that readers activate IC information at the beginning of the subordinate clause or even at the verb itself. Studies across different languages and learners have confirmed this perspective. For example, research with Dutch natives [6,10], Finnish natives [8], Chinese natives [11], Korean English learners [3], and Chinese English learners [15] all support the notion that readers can activate verb-based IC information quickly, even before encountering the disambiguating information during the interpretation of a pronoun in a sentence or discourse.

The current study provides evidence on the use of verb-based IC information during L2 learners’ pronoun anaphoric inference. It proposes that similar to native speakers and other L2 learners, Chinese English learners can also activate verb-based IC information soon after the verb is processed. This activation occurs before any disambiguating information is encountered, aligning with the focusing account [10,13,24,26,33].

### 4.2. The Influence of L2 Proficiency

The results indicate that L2 proficiency significantly impacts Chinese English learners’ pronoun anaphoric inference. Specifically, the high-proficiency group required less processing time compared to the low-proficiency group. This effect was observed on RPD at the critical pronoun and on FGD, RPD, and TRT at the critical region. Successful pronoun assignment relies on the effective use of various resources. In light of Kaan’s [42] (p. 268) discovery that “various studies have shown that predictive behavior increases with increasing proficiency”, investigating the effect of L2 proficiency on pronoun anaphoric inference is crucial. However, Kim and Grüter [3] did not find a significant influence of L2 proficiency on pronoun anaphoric inference. They suggested that this might be due to “limited variance in proficiency among the sample investigated, lack of sensitivity of the specific proficiency measures employed, and reduced power due to limited sample size” (p. 18). In contrast, the current study did find a significant effect of L2 proficiency. This discrepancy could be attributed to differences in the L2 learners studied (e.g., Korean English learners vs. Chinese English learners), the experimental paradigms used (e.g., visual world vs. normal sentence reading), or the materials employed.

The finding that the high-proficiency group required less processing time than the low-proficiency group can be explained by the following aspects. First, high-proficiency Chinese English learners are more adept at processing linguistic information due to their better command of grammar, vocabulary, and structural knowledge of English. This allows them to quickly integrate different types of information (like lexical, syntactic, and discourse-level cues) to make pronoun anaphoric inferences. Specifically, when high-proficiency learners encounter a pronoun, they can rapidly retrieve potential antecedents and assess their compatibility based on number, gender, and personal features. Due to their familiarity with L2 structures, high-proficiency learners are more likely to anticipate the upcoming linguistic elements (such as the antecedent of a pronoun) and confirm or adjust these predictions quickly. Lower proficiency learners often require more reanalysis when their initial parsing is incorrect. High-proficiency learners, on the other hand, make more accurate predictions in advance, reducing the need for reanalysis. Second, the RAGE hypothesis [17,18] suggests that L2 learners are generally less likely to generate expectations during sentence processing compared to native speakers. High-proficiency L2 learners exhibit a significantly improved ability to generate and use expectations compared to their low-proficiency counterparts. For low-proficiency learners, they need to process linguistic information incrementally, without the benefit of making strong predictions. This leads to slower processing as they wait for full disambiguation to occur. They may rely more heavily on bottom-up processing, parsing each part of the sentence before concluding. This slower, less anticipatory approach increases the time needed for making pronoun anaphoric inference. In contrast, high-proficiency learners can use verb-based IC information and other linguistic cues to anticipate the referent of a pronoun, which significantly speeds up the processing of anaphoric references. Their ability to anticipate and confirm these expectations means they spend less time interpreting the pronoun, as they are already prepared for the potential referents. Third, cognitive load can also affect processing speed. Low-proficiency learners face more cognitive load because they need to spend additional time understanding sentence structures, accessing their lexicon, and integrating context. This added burden slows down their ability to make pronoun anaphoric inferences. High-proficiency learners, on the other hand, have better-developed cognitive resources for processing the language, allowing them to devote more attention to making pronoun anaphoric inferences quickly.

Thus, the faster processing time for high-proficiency L2 learners in pronoun anaphoric inference tasks is a reflection of their improved ability to predict and integrate linguistic information. Their proficiency reduces the cognitive load associated with processing and allows them to anticipate referents more effectively. In contrast, low-proficiency learners are hampered by weaker predictive abilities and a greater reliance on bottom-up processing, leading to slower resolution of pronoun references. The RAGE hypothesis provides a useful framework for understanding these differences, as it highlights the role of expectation generation in L2 learners’ language processing.

### 4.3. The Joint Influence of Verb-Based IC Information and L2 Proficiency

The interactive influence of verb-based IC information and L2 proficiency was evident both at the critical pronoun and critical region. At the critical pronoun, significant two-way interactions were observed between L2 proficiency and congruency, as well as between verb bias and congruency, with both interactions affecting RPD. At the critical region, the interaction between L2 proficiency and congruency was also significant. Additionally, significant differences were found in the two-way interaction between verb bias and congruency and the three-way interaction among L2 proficiency, verb bias, and congruency, affecting RPD and TRT, respectively. Notably, all interactive effects aligned with the assumptions except for the three-way interaction among L2 proficiency, verb bias, and congruency at RPD. Further analysis revealed that under the NP2-biased condition, high-proficiency learners took a longer time to assign a pronoun to an antecedent in the congruent condition compared to in the incongruent condition. To explain the contrast in the NP2-biased condition where high-proficiency learners took longer to assign a pronoun to an antecedent in the congruent condition than in the incongruent condition, several possibilities could be considered. Firstly, high-proficiency learners tend to rely more heavily on predictive mechanisms during reading [17,18]. They might have anticipated that the congruent condition would involve easier or faster pronoun resolution, but the actual processing could have been delayed by their heightened expectations. In the congruent condition, their strong expectation of a particular outcome (i.e., assigning the pronoun to the expected NP2 antecedent) may have paradoxically slowed their processing. This is likely because they were checking for confirmatory cues to ensure that their prediction was correct. This increased cognitive load, driven by self-monitoring and confirmatory processing, may have resulted in longer reading times, even though the condition was congruent with their expectations. That is, when their predictions were too strong, it could have taken time longer for them to confirm and resolve the pronoun, hence the delay in the congruent condition. Secondly, in the incongruent condition, the conflict between the bias and the actual antecedent may have triggered immediate reanalysis, allowing learners to adjust quickly to the incongruency. However, in the congruent condition, the ease of assignment might have led them to initially rely too much on automatic processes, delaying final resolution as they checked for more confirmatory cues, increasing total processing times. Thirdly, the focusing account suggests that verb-based IC information could activate expectations early in the sentence, but for high-proficiency learners, this activation might have caused them to second-guess their immediate resolution of the pronoun in congruent cases. This self-monitoring or secondary check could explain the longer processing time. In incongruent cases, the contradiction might have been more noticeable, prompting faster processing. Fourthly, another possible explanation is that other sentence-level factors such as sentence complexity or ambiguity could be at play. If the congruent condition involved additional information or structural features that subtly increased processing difficulty (even if the overall bias was congruent), this could explain the slower processing time than in the incongruent condition. All in all, we believe that these factors—particularly the interaction between predictive processing and reanalysis—provide a plausible explanation for the longer reading times in the congruent condition. However, we acknowledge that this finding deserves further investigation in future research to better understand the relationship between language proficiency, prediction, and pronoun anaphoric inference.

## 5. General Discussion

This study investigated the influence of verb-based IC information and L2 proficiency on Chinese English learners’ pronoun anaphoric inference. The results revealed significant main effects for all three influencing factors. At the critical pronoun, L2 proficiency significantly affected RPD, verb bias significantly affected TRT, and congruency significantly affected both RPD and TRT. Moreover, the effects of two-way interactions between L2 proficiency and congruency and between verb bias and congruency were significant on RPD. At the critical region, L2 proficiency had significant effects on FGD, RPD, and TRT, while verb bias significantly impacted TRT, and congruency significantly affected RPD and TRT. Additionally, the effect of the two-way interaction between L2 proficiency and verb bias was significant on PRD. A significant two-way interaction between verb bias and congruency was observed, affecting both RPD and TRT. The three-way interaction among verb bias, congruency, and L2 proficiency also showed a significant effect on RPD and TRT.

The first purpose of this research was to explore the activation of verb-based IC information on Chinese English learners’ pronoun anaphoric inference. The results indicated that Chinese English learners could actively use verb-based IC information at both the critical pronoun and critical region before encountering disambiguating information, aligning with the focusing account [10,13,24,26,33]. A central feature in discourse comprehension is the link between discourse focus and readers’ prediction of what will be mentioned in the subsequent part. According to Levy’s [56] (p. 1128) surprisal theory, “surprisal serves as a causal bottleneck between the linguistic representations constructed during sentence comprehension and the processing difficulty incurred at a given word within a sentence”. That is to say, the difficulty in sentence comprehension is caused by the surprisal of the incoming word. In pronoun anaphoric inference, when readers encounter the biased verb, he or she will make predictions about who is more likely to be re-mentioned in the forthcoming discourse. A following congruent pronoun will result in low surprisal, and a subsequent incongruent pronoun will bring about high surprisal. Thus, processing a sentence with an incongruent pronoun takes a longer time than processing one with a congruent pronoun. Although verb-based IC information significantly impacts Chinese English learners’ pronoun anaphoric inference and they generally adopt the recency strategy, highly proficient learners sometimes exhibit first mention or subject preference [51,52,53,54], treating the subject as the antecedent of the pronoun.

The second purpose was to examine the impact of L2 proficiency on pronoun anaphoric inference. The findings replicate previous research demonstrating the influence of individual differences on this process [3,9,13,14,15,41]. High-proficiency L2 learners demonstrate faster processing times in making pronoun anaphoric inference due to their enhanced capacity to predict and integrate linguistic cues. Their higher proficiency enables them to efficiently reduce cognitive load during processing, allowing them to anticipate pronoun referents with greater accuracy. They are more adept at predicting who is likely to be re-mentioned in the upcoming discourse and at using the provided information to make accurate causal inference. In contrast, low-proficiency learners struggle with weaker predictive skills and rely more heavily on bottom-up processing, which slows down their ability to resolve pronouns. The RAGE hypothesis [17,18] helps explain these differences by emphasizing the reduced ability of L2 learners to generate expectations during language processing, particularly among those with lower proficiency.

Although the current study did not observe significant effects in the earliest eye movement measures, namely FFD and FGD, the significant findings on RPD and TRT still provide strong support for the predictions of the focusing account. The focusing account posits that verb-based IC information influences sentence processing early, which may lead to a delay in comprehension. While early eye movement measures like FFD and FGD are often associated with initial lexical processing, the absence of effects in these measures does not undermine the validity of the observed effects in later processing stages. The focusing account emphasizes that the processing delay occurs at an early point in sentence comprehension. However, it is important to recognize that “early” in this context does not necessarily equate to the earliest possible eye movement measures (e.g., FFD and FGD). Instead, the processing delay predicted by the focusing account may manifest slightly later in more complex measures, such as RPD or TRT. These later measures can more effectively capture subtle cognitive processes related to the integration of IC information, especially in tasks involving complex pronoun anaphoric inference in L2. Therefore, the significant effects observed in these later measures at the critical pronoun and region are still consistent with the predictions of the focusing account. They demonstrate that IC information affects sentence comprehension as anticipated, even if these effects do not appear immediately in the earliest eye movement stages. These findings suggest that the cognitive load involved in resolving IC information may extend beyond initial lexical processing, leading to processing delays detectable at later stages of sentence comprehension. In light of this, we contend that the significant results on PRD and TRT offer robust evidence in support of the focusing account, even though early eye movement measures did not show significant differences.

This result may also suggest that the reading patterns of Chinese English learners do not fully conform to either the focusing account [10,13,24,26,33] or the integration account [12,32,34,35], but rather reflect a mixed processing model. In this mixed account, L2 learners may engage in both early activation of verb-based implicit causality (IC) information, as suggested by the focusing account, while also relying on later-stage information integration, as emphasized in the integration account. Specifically, while L2 learners may activate IC information early in the sentence, this activation could be less robust or more delayed than native speakers due to the additional cognitive demands of processing a second language. As a result, some aspects of information may only be fully integrated at the later stages of sentence processing. This hybrid pattern suggests that L2 learners might be drawing on both early focusing and later integration mechanisms, influenced by factors such as their language proficiency and the complexity of the linguistic input. Such findings indicate a need for further investigation into how L2 learners balance these different processing strategies; how language proficiency and linguistic context influence the interaction between early and late-stage sentence comprehension processes; and how the timing of these effects varies depending on task complexity, language proficiency, and the nature of the IC bias.

In conclusion, this study shows that both high- and low-proficiency Chinese English learners can activate verb-based IC information at the critical pronoun and critical region. The findings suggest that, like native speakers, Chinese English learners can rapidly utilize verb-based IC information for pronoun anaphoric inference, supporting the focusing account [10,13,24,26,33]. Additionally, L2 proficiency influences this process, with high-proficiency readers making pronoun anaphoric inferences more quickly than their lower-proficiency counterparts.

The current study is important for several reasons. First, it deepens our understanding of how Chinese English learners, as L2 speakers, make pronoun anaphoric inferences during reading, particularly in light of verb-based IC information. By investigating this cognitive process through eye-tracking, this study provides real-time insights into how L2 learners navigate complex linguistic cues in L2. Second, this study extends research on IC by examining how verb-specific expectations influence sentence processing. By focusing on Chinese English learners, it adds cross-linguistic evidence to IC-related studies, offering new perspectives on how IC information is used differently in non-native language contexts. Third, this research sheds light on the role of L2 proficiency in affecting the use of IC information during pronoun anaphoric inference. It contributes to our knowledge of how varying levels of L2 proficiency affect cognitive strategies, such as prediction and integration, in language comprehension. Fourth, adopting the normal reading paradigm in eye tracking allows for a nuanced, moment-by-moment analysis of reading patterns. This method provides precise measurements of cognitive processing, helping to identify subtle differences in how learners at different proficiency levels handle pronoun anaphoric inference and linguistic expectations. Fifth, the findings have practical implications for language teaching, particularly in how verb-based IC cues can be integrated into L2 reading instruction. Understanding the cognitive processes involved in pronoun resolution could inform teaching strategies that improve L2 learners’ comprehension skills. Last, this study addresses a gap in L2 processing research, particularly concerning how verb-based IC information and L2 proficiency interact to shape language processing. It also offers empirical evidence that enhances theoretical models, such as the RAGE hypothesis [17,18], by connecting them to eye movement patterns in L2 learners. Overall, this study is significant for advancing both theoretical and practical knowledge about L2 processing, verb-based IC information, and the cognitive mechanisms that underpin pronoun anaphoric inference in L2 learners.

## 6. Conclusions

This study examines the impact of verb-based IC information and L2 proficiency on Chinese English learners’ pronoun anaphoric inference using a normal sentence reading paradigm in eye-tracking studies. The results indicate that Chinese English learners can utilize verb-based IC information at an early stage, aligning with the focusing account [10,13,24,26,33]. Additionally, L2 proficiency accounts for variability in inference, with high-proficiency learners requiring less time than their low-proficiency counterparts. The findings from this study provide several important practical implications, particularly for L2 acquisition and teaching. Firstly, this study sheds light on how verb-based IC information influences sentence processing, especially in pronoun anaphoric inference among L2 learners. This suggests that teachers should place more emphasis on IC verbs and their roles in discourse comprehension. By incorporating IC verbs into L2 curricula, educators can help learners develop better predictive strategies, which are essential for efficient sentence processing. Explicitly teaching the role of IC biases in sentence comprehension could improve students’ ability to anticipate and make pronoun anaphoric inference in complex sentence structures, which is particularly useful for learners of languages like English, where such biases are important. Furthermore, this study highlights the influence of L2 proficiency on pronoun anaphoric inference, suggesting that more proficient L2 learners might be better at generating expectations based on verb-based IC information. This finding could guide the development of proficiency assessments that include measures of predictive processing ability. It also suggests that teaching interventions should consider learners’ language proficiency levels, offering more focused practice on verb-based IC information comprehension for less proficient learners. Moreover, this study, by focusing on Chinese English learners, suggests that verb-based IC information plays a significant role across languages with different syntactic and morphological structures. This finding has implications for the generalization of IC processing mechanisms to other L1–L2 pairings and highlights the need for more cross-linguistic research to further explore how different language structures influence sentence processing.

While the study offers valuable insights, it also has several limitations that should be acknowledged. Firstly, one major limitation is the absence of a native speaker control group. Without this baseline, it is difficult to determine whether the observed processing patterns are unique to L2 learners or whether they reflect general IC processing mechanisms common to both L1 and L2 speakers. Future research should include native English speakers as a control group to better assess whether the findings are specific to L2 learners. Secondly, this study focused exclusively on Chinese L2 learners of English, which may limit the generalizability of the findings to learners from other linguistic backgrounds. Different L1 speakers may process IC information differently depending on the syntactic and morphological characteristics of their native languages. This suggests the need for further studies involving learners from other L1 backgrounds to verify whether the findings hold across diverse populations. Thirdly, pronoun anaphoric inference involves complex interactions between verb biases, discourse context, and referential resolution. The study does not fully explore how these interactions play out in more naturalistic, conversational contexts, where discourse factors such as topic continuity and world knowledge can further influence pronoun anaphoric inference. Future research could adopt more ecologically valid experimental designs to better capture the full range of factors involved in IC processing. While the findings are exploratory, many more studies are needed to further examine the influence of verb-based IC information during pronoun anaphoric inference as well as the effect of some other factors, especially for L2 learners.

## Figures and Tables

**Table 1:** Verbs used in the experiment with their bias and familiarity ratings.

NP1-Biased Verb	Bias	Familiarity	NP2-Biased Verb	Bias	Familiarity
lied to	1.16	6.49	appreciated	1.95	6.10
hurt	1.19	4.92	punished	1.90	6.39
called	1.20	6.44	admired	1.89	6.08
bothered	1.23	5.23	laughed at	1.86	5.79
pained	1.25	5.26	congratulated	1.85	4.64
troubled	1.26	5.97	employed	1.83	6.00
delighted	1.30	5.50	worried about	1.81	6.34
cheated	1.31	6.24	celebrated	1.81	6.49
telephoned	1.31	5.92	thanked	1.80	6.42
apologized to	1.33	5.47	disliked	1.79	6.21
exhausted	1.33	6.10	rewarded	1.78	5.79
attracted	1.35	5.82	praised	1.76	5.62
shocked	1.35	6.32	prized	1.74	5.11
annoyed	1.36	6.13	corrected	1.74	5.95

**Table 2:** Statistical analysis results of different eye movement measures at the critical pronoun.

	Estimate	S.E.	df	t	*p*
First Fixation Durations					
(Intercept)	5.361	0.022	212.700	242.309	<0.001 ***
L2 Proficiency	0.014	0.031	264.000	0.464	0.643
Verb Bias	−0.004	0.024	264.100	−0.181	0.857
Congruency	0.021	0.023	245.000	0.902	0.368
L2 Proficiency × Verb Bias	−0.015	0.034	407.500	−0.455	0.649
L2 Proficiency × Congruency	−0.015	0.031	2472.000	−0.493	0.622
Verb Bias × Congruency	−0.016	0.033	400.000	−0.504	0.615
L2 Proficiency × Verb Bias × Congruency	0.023	0.045	2512.000	0.506	0.613
First Gaze Durations					
(Intercept)	5.373	0.022	269.600	242.158	<0.001 ***
L2 Proficiency	0.031	0.032	448.100	0.973	0.331
Verb Bias	−0.004	0.025	385.500	−0.148	0.883
Congruency	0.036	0.024	227.300	1.490	0.138
L2 Proficiency × Verb Bias	0.021	0.035	2549.000	0.607	0.544
L2 Proficiency × Congruency	−0.026	0.034	2553.000	−0.775	0.439
Verb Bias × Congruency	−0.013	0.035	351.500	−0.370	0.712
L2 Proficiency × Verb Bias × Congruency	−0.005	0.049	2543.000	−0.096	0.924
Regression Path Durations					
(Intercept)	5.525	0.031	251.553	177.245	<0.001 ***
L2 Proficiency	0.077	0.044	634.491	1.745	0.081.
Verb Bias	−0.011	0.038	370.337	−0.293	0.770
Congruency	0.141	0.037	228.487	3.756	<0.001 ***
L2 Proficiency × Verb Bias	0.022	0.054	2546.342	0.408	0.683
L2 Proficiency × Congruency	−0.134	0.053	2548.014	−2.528	0.012 *
Verb Bias × Congruency	−0.170	0.054	352.844	−3.156	0.002 **
L2 Proficiency × Verb Bias × Congruency	0.105	0.076	2538.741	1.382	0.167
Total Reading Times					
(Intercept)	5.684	0.044	216.000	129.868	<0.001 ***
L2 Proficiency	0.087	0.056	233.300	1.553	0.122
Verb Bias	−0.125	0.046	504.900	−2.709	0.007 **
Congruency	0.110	0.044	535.400	2.494	0.013 *
L2 Proficiency × Verb Bias	−0.015	0.059	421.400	−0.248	0.804
L2 Proficiency × Congruency	−0.008	0.053	2462.000	−0.146	0.884
Verb Bias × Congruency	−0.097	0.061	945.300	−1.596	0.111
L2 Proficiency × Verb Bias × Congruency	0.060	0.076	2499.000	0.796	0.426

Note: (1) Formula for each model: lmer (logRT~Verb Bias × Congruency × L2 Proficiency + (1|Participant) + (1|Item)); (2) ***: *p* < 0.001, **: *p* < 0.01; *: *p* < 0.05.

**Table 3:** Statistical analysis results of different eye movement measures at the critical region.

	Estimate	S.E.	df	t	*p*
First Fixation Durations					
(Intercept)	5.449	0.022	241.865	250.378	<0.001 ***
L2 Proficiency	0.050	0.031	299.819	1.603	0.110
Verb Bias	0.013	0.021	365.675	0.655	0.513
Congruency	−0.022	0.021	229.685	−1.087	0.278
L2 Proficiency × Verb Bias	−0.047	0.029	3716.756	−1.614	0.107
L2 Proficiency × Congruency	−0.024	0.029	3716.005	−0.804	0.421
Verb Bias × Congruency	−0.016	0.029	358.658	−0.560	0.576
L2 Proficiency × Verb Bias × Congruency	0.055	0.041	3717.137	1.326	0.185
First Gaze Durations					
(Intercept)	5.849	0.031	195.800	188.955	<0.001 ***
L2 Proficiency	0.122	0.045	234.200	2.735	0.007 **
Verb Bias	0.020	0.029	334.100	0.691	0.490
Congruency	0.023	0.032	198.700	0.712	0.477
L2 Proficiency × Verb Bias	0.002	0.042	3626.000	0.039	0.969
L2 Proficiency × Congruency	−0.020	0.046	394.500	−0.440	0.660
Verb Bias × Congruency	−0.058	0.042	325.800	−1.404	0.161
L2 Proficiency × Verb Bias × Congruency	0.027	0.059	3626.000	0.459	0.646
Regression Path Durations					
(Intercept)	6.001	0.039	257.932	155.391	<0.001 ***
L2 Proficiency	0.312	0.054	280.293	5.775	<0.001 ***
Verb Bias	0.038	0.035	603.304	1.099	0.272
Congruency	0.096	0.036	333.059	2.672	0.008 **
L2 Proficiency × Verb Bias	−0.105	0.047	3734.166	−2.230	0.026 *
L2 Proficiency × Congruency	−0.066	0.047	3734.357	−1.397	0.163
Verb Bias × Congruency	−0.169	0.049	575.225	−3.422	<0.001 ***
L2 Proficiency × Verb Bias × Congruency	0.150	0.067	3734.073	2.247	0.025 *
Total Reading Times					
(Intercept)	6.447	0.048	238.669	133.084	<0.001 ***
L2 Proficiency	0.220	0.064	221.544	3.428	<0.001 ***
Verb Bias	−0.114	0.037	1631.412	−3.086	0.002 **
Congruency	0.233	0.039	868.982	5.952	<0.001 ***
L2 Proficiency × Verb Bias	−0.060	0.047	3750.469	−1.285	0.199
L2 Proficiency × Congruency	−0.080	0.047	3750.450	−1.715	0.086.
Verb Bias × Congruency	−0.219	0.052	1509.581	−4.183	<0.001 ***
L2 Proficiency × Verb Bias × Congruency	0.143	0.066	3750.348	2.167	0.030 *

Note: (1) Formula for each model: lmer (logRT~Verb Bias × Congruency × L2 Proficiency + (1|Participant) + (1|Item)); (2) ***: *p* < 0.001, **: *p* < 0.01; *: *p* < 0.05.

## Data Availability

The data presented in this study are available on request from the corresponding author. The data are not publicly available due to privacy concerns.

## References

[1] Cheng W., Almor A. (2019). A Bayesian approach to establishing coreference in second language discourse: Evidence from implicit causality and consequentiality verbs. Biling.-Lang. Cogn..

[2] Levine W.H., Guzmán A.E., Klin C.M. (2000). When anaphor resolution fails. J. Mem. Lang..

[3] Kim H., Grüter T. (2021). Predictive processing of implicit causality in a second language: A visual-world eye-tracking study. Stud. Second Lang. Acquis..

[4] Rudolph U., Försterling F. (1997). The psychological causality implicit in verbs: A review. Psychol. Bull..

[5] Garvey C., Caramazza A. (1974). Implicit causality in verbs. Linguist. Inq..

[6] Cozijn R., Commandeur E., Vonk W., Noordman L.G. (2011). The time course of the use of implicit causality information in the processing of pronouns: A visual world paradigm study. J. Mem. Lang..

[7] Featherstone C.R., Sturt P. (2010). Because there was a cause for concern: An investigation into a word-specific prediction account of the implicit-causality effect. Q. J. Exp. Psychol..

[8] Järvikivi J., Van Gompel R.P.G., Hyönä J. (2017). The interplay of implicit causality, structural heuristics, and anaphor type in ambiguous pronoun resolution. J. Psycholinguist. Res..

[9] Koornneef A., Dotlačil J., van den Broek P., Sanders T. (2016). The influence of linguistic and cognitive factors on the time course of verb-based implicit causality. Q. J. Exp. Psychol..

[10] Koornneef A., Van Berkum J.J.A. (2006). On the use of verb-based implicit causality in sentence comprehension: Evidence from self-paced reading and eye tracking. J. Mem. Lang..

[11] Lyu S., Wang L. (2022). Implicit causality and pronoun resolution in intersubjective discourse relations. Front. Psychol..

[12] Stewart A.J., Pickering M.J., Sanford A.J. (2000). The time course of the influence of implicit causality information: Focusing versus integration accounts. J. Mem. Lang..

[13] Long D.L., De Ley L. (2000). Implicit causality and discourse focus: The interaction of text and reader characteristics in pronoun resolution. J. Mem. Lang..

[14] Van Berkum J.J.A., De Goede D., Van Alphen P.M., Mulder E.R., Kerstholt J.H. (2013). How robust is the language architecture? The case of mood. Front. Psychol..

[15] Wang T., Gabriele A. (2023). Individual differences modulate sensitivity to implicit causality bias in both native and nonnative processing. Stud. Second Lang. Acquis..

[16] Kim H., Grüter T. (2019). Cross-linguistic activation of implicit causality biases in Korean learners of English. Biling.-Lang. Cogn..

[17] Grüter T., Rohde H., Schafer A.J., Orman W., Valleau M.J. (2014). The role of discourse-level expectations in non-native speakers’ referential choices. Proceedings of the 38th Annual Boston University Conference on Language Development.

[18] Grüter T., Rohde H., Schafer A.J. (2017). Coreference and discourse coherence in L2. Linguist. Approaches Biling..

[19] Kehler A., Kertz L., Rohde H., Elman J.L. (2008). Coherence and coreference revisited. J. Semant..

[20] Au T.K. (1986). A verb is worth a thousand words: The causes and consequences of interpersonal events implicit in language. J. Mem. Lang..

[21] Bott O., Solstad T. (2021). Discourse expectations: Explaining the implicit causality biases of verbs. Linguistics.

[22] Brown R., Fish D. (1983). The psychological causality implicit in language. Cognition.

[23] Garnham A., Oakhill J., Cruttenden H. (1992). The role of implicit causality and gender cue in the interpretation of pronouns. Lang. Cogn. Process..

[24] Greene S.B., McKoon G. (1995). Telling something we can’t know: Experimental approaches to verbs exhibiting implicit causality. Psychol. Sci..

[25] Hartshorne J.K. (2014). What is implicit causality?. Lang. Cogn. Neurosci..

[26] McKoon G., Greene S.B., Ratcliff R. (1993). Discourse models, pronoun resolution, and the implicit causality of verbs. J. Exp. Psychol.-Learn. Mem. Cogn..

[27] Solstad T., Bott O. (2022). On the nature of implicit causality and consequentiality: The case of psychological verbs. Lang. Cogn. Neurosci..

[28] Van Berkum J.J.A., Koornneef A.W., Ottena M., Nieuwland M.S. (2007). Establishing reference in language comprehension: An electrophysiological perspective. Brain Res..

[29] Sun Y., Shu H., Zhou X., Zhen X. (2001). The effect of implicit verb causality on pronoun processing. Psychol. Sci..

[30] Ferstl E.C., Garnham A., Manouilidou C. (2011). Implicit causality bias in English: A corpus of 300 verbs. Behav. Res. Methods.

[31] Pyykkönen P., Järvikivi J. (2010). Activation and persistence or implicit causality information in spoken language comprehension. Exp. Psychol..

[32] Zhang X., Bai X., Yan G. (2006). The role and time course of verbs’ implicit causality in pronoun resolution. Psychol. Sci..

[33] McDonald J.L., MacWhinney B. (1995). The time course of anaphor resolution: Effects of implicit verb causality and gender. J. Mem. Lang..

[34] Bai X., Zhang X., Yan G. (2005). The eye movement research on the time course of verb’s implicit causality in pronoun resolution. Psychol. Explor..

[35] Garnham A., Traxler M., Oakhill J., Gernsabacher M.A. (1996). The locus of implicit causality effects in comprehension. J. Mem. Lang..

[36] Cheng W., Almor A. (2017). The effect of implicit causality and consequentiality on nonnative pronoun resolution. Appl. Psycholinguist..

[37] Contemori C., Dussias P.E., Bertolini A.B., Kaplan M.J. (2018). Prediction at the discourse level in L2 English speakers: An eye-tracking study. Proceedings of the 42nd Annual Boston University Conference on Language Development.

[38] Contemori C., Dussias P.E. (2019). Prediction at the discourse level in Spanish-English bilinguals: An eye-tracking study. Front. Psychol..

[39] Liu R., Nicol J., Prior M.T., Watanabe Y., Lee S.-K. (2010). Online processing of anaphora by advanced English learners. Selected Proceedings of the 2008 Second Language Research Forum, Honolulu, HI, USA, 17–19 October 2008.

[40] Wu M., Wu D. (2019). The effect of implicit causality of L2 verbs on the antecedent assignment of pronouns. For. Lang. China.

[41] Johnson E., Arnold J.E. (2021). Individual differences in print exposure predict use of implicit causality in pronoun comprehension and referential prediction. Front. Psychol..

[42] Kaan E. (2014). Predictive sentence processing in L2 and L1: What is different?. Linguist. Approaches Biling..

[43] Chambers C.G., Cooke H. (2009). Lexical competition during second-language listening: Sentence context, but not proficiency, constrains interference from the native lexicon. J. Exp. Psychol.-Learn. Mem. Cogn..

[44] Dussias P.E., Valdés Kroff J.R., Guzzardo Tamargo R.E., Gerfen C. (2013). When gender and looking go hand in hand: Grammatical gender processing in L2 Spanish. Stud. Second Lang. Acquis..

[45] Hopp H. (2013). Grammatical gender in adult L2 acquisition: Relations between lexical and syntactic variability. Second Lang. Res..

[46] Garnham A., Vorthmann S., Kaplanova K. (2021). Implicit consequentiality bias in English: A corpus of 300+ verbs. Behav. Res. Methods.

[47] Koornneef A.W., Sanders T.J.M. (2013). Establishing coherence relations in discourse: The influence of implicit causality and connectives on pronoun resolution. Lang. Cogn. Process..

[48] Box G.E.P., Cox D.R. (1964). An analysis of transformations. J. R. Stat. Soc. Ser. B-Stat. Methodol..

[49] Bates D., Mächler M., Bolker B.M., Walker S.C. (2015). Fitting linear mixed-effects models using lme4. J. Stat. Softw..

[50] Lenth R.V., Bolker B., Buerkner P., Giné-Vázquez I., Herve M., Jung M., Love J., Miguez F., Riebl H., Singmann H. (2023). *Emmeans: Estimated Marginal Means, Aka Least-Squares Means* (Version 1.8.9) [R Package]. https://CRAN.R-project.org/package=emmeans.

[51] Crawley R.A., Stevenson R.J., Kleinman D. (1990). The use of heuristic strategies in the interpretation of pronouns. J. Psycholinguist. Res..

[52] Gernsbacher M.A., Hargreaves D.J. (1988). Accessing sentence participants: The advantage of first mention. J. Mem. Lang..

[53] Gernsbacher M.A., Hargreaves D.J., Beeman M. (1989). Building and accessing clausal representations—The advantage of first mention versus the advantage of clause recency. J. Mem. Lang..

[54] Järvikivi J., Van Gompel R.P.G., Hyönä J., Bertram R. (2005). Ambiguous pronoun resolution: Contrasting the first-mention and subject-preference accounts. Psychol. Sci..

[55] Ehrlich K., Rayner K. (1983). Pronoun assignment and semantic integration during reading: Eye movements and immediacy of processing. J. Verbal Learn. Verbal Behav..

[56] Levy R. (2008). Expectation-based syntactic comprehension. Cognition.

